# Alterations in proton leak, oxidative status and uncoupling protein 3 content in skeletal muscle subsarcolemmal and intermyofibrillar mitochondria in old rats

**DOI:** 10.1186/1471-2318-14-79

**Published:** 2014-06-21

**Authors:** Raffaella Crescenzo, Francesca Bianco, Arianna Mazzoli, Antonia Giacco, Giovanna Liverini, Susanna Iossa

**Affiliations:** 1 Department of Biology, Complesso Universitario di Monte Sant'Angelo, Edificio 7, Via Cinthia, I-80126 Napoli, Italy

**Keywords:** Mitochondria, Aging, Oxidative stress

## Abstract

**Background:**

We considered of interest to evaluate how aging affects mitochondrial function in skeletal muscle.

**Methods:**

We measured mitochondrial oxidative capacity and proton leak, together with lipid oxidative damage, superoxide dismutase specific activity and uncoupling protein 3 content, in subsarcolemmal and intermyofibrillar mitochondria from adult (six months) and old (two years) rats. Body composition, resting metabolic rate and plasma non esterified fatty acid levels were also assessed.

**Results:**

Old rats displayed significantly higher body energy and lipids, while body proteins were significantly lower, compared to adult rats. In addition, plasma non esterified fatty acid levels were significantly higher, while resting metabolic rates were found to be significantly lower, in old rats compared to adult ones. Significantly lower oxidative capacities in whole tissue homogenates and in intermyofibrillar and subsarcolemmal mitochondria were found in old rats compared to adult ones. Subsarcolemmal and intermyofibrillar mitochondria from old rats exhibited a significantly lower proton leak rate, while oxidative damage was found to be significantly higher only in subsarcolemmal mitochondria. Mitochondrial superoxide dismutase specific activity was not significantly affected in old rats, while significantly higher content of uncoupling protein 3 was found in both mitochondrial populations from old rats compared to adult ones, although the magnitude of the increase was lower in subsarcolemmal than in intermyofibrillar mitochondria.

**Conclusions:**

The decrease in oxidative capacity and proton leak in intermyofibrillar and subsarcolemmal mitochondria could induce a decline in energy expenditure and thus contribute to the reduced resting metabolic rate found in old rats, while oxidative damage is present only in subsarcolemmal mitochondria.

## Background

Aging is usually defined as a time-dependent decline of maximal functionality that affects tissues and organs of the whole body and leads to an increased susceptibility to disease and risk of death [1]. Post-mitotic tissues such as skeletal muscle are preferentially prone to the adverse effects of advancing age [2]. It has also been pointed out that mitochondria are a key factor in this process [3,4], since they have a major role in energetic homeostasis so determining ATP availability in the cells. Indeed, dysfunctional mitochondria will be unable to meet cellular ATP demands, thus compromising the cellular ability to adapt to physiological stress imposed to skeletal muscle across the entire lifespan [5].

It should be taken into account that ATP production following the oxidation of metabolic fuels depends on oxidative capacity and energetic efficiency of the mitochondrial compartment. Changes in each of these factors could theoretically affect mitochondrial bioenergetics and should be monitored. In addition, skeletal muscle mitochondrial population is heterogeneous, composed of mitochondria located either beneath the sarcolemmal membrane (subsarcolemmal) or between the myofibrils (intermyofibrillar) [6]. Since these two mitochondrial populations exhibit different energetic characteristics and therefore can be differently affected by physiological stimuli [7], it is important that both are separately studied. However, to our knowledge, studies carried out on both mitochondrial populations from skeletal muscle during aging are scarce [8,9].

We have previously found that, in the transition from young (60 days) to adult (180 days) age, there is an increase in energetic efficiency in subsarcolemmal and intermyofibrillar skeletal muscle mitochondria [10]. This modification in mitochondrial performance occurred concomitantly with an increase in whole body lipids and plasma non esterified fatty acids (NEFA), suggesting a link between skeletal muscle mitochondrial efficiency and metabolic impairments [10]. We therefore considered of interest to extend previous results by evaluating how the progression of the aging affects skeletal muscle mitochondrial function. To this end, after whole body metabolic characterization, obtained by body composition, resting metabolic rate (RMR) and plasma NEFA determination, mitochondrial respiratory capacity and proton leak (an index of mitochondrial energetic efficiency) in subsarcolemmal and intermyofibrillar mitochondria from adult (six months) and old (two years) rats were assessed. Mitochondrial lipid peroxidation was also determined in skeletal muscle from adult and old rats, to evaluate mitochondrial oxidative damage. Finally, skeletal muscle antioxidant defense was assessed by measuring superoxide dismutase (SOD) specific activity and uncoupling protein 3 (UCP3) content, since it has been demonstrated that this protein plays a role in the defence of mitochondria from oxidative damage [11].

## Methods

### Ethical approval

Treatment, housing, and killing of rats met the guidelines set by the Italian Health Ministry. All experimental procedures involving rats were approved by “Comitato Etico-Scientifico per la Sperimentazione Animale” of the University “Federico II” of Naples.

Male Wistar rats were housed at 24°C under an artificial circadian 12 h light/12 h dark cycle and received ad libitum standard stock diet and water. Two groups of 8 rats each were used, aged 6 months (adult rats) or 24 months (old rats).

### RMR measurement

RMR was measured in all rats in the morning with a four-chamber indirect open-circuit calorimeter (Panlab s.r.l., Cornella, Barcelona, Spain). Although most rats quieted down after 30 min in the chamber, all rats were allowed to adapt to the experimental environment for a minimum of 60 min before beginning the measurements. RMR was measured in the fed state and the day after in food-deprived rats in a chamber at 24°C over a period of at least 10 min during which the rat remained quiet.

### Body composition

The rats were subjected to euthanasia, blood and hind leg skeletal muscles were harvested, and the carcasses were used for body composition determination.

Guts were cleaned of undigested food and the carcasses were then autoclaved. After dilution in distilled water and subsequent homogenisation of the carcasses with a Polytron homogeniser (Kinematica, Switzerland), duplicate samples of the homogenised carcass were analyzed for energy content by bomb calorimetry. To take into account the energy content of skeletal muscle, tissue samples were dried and the energy content was then measured with the bomb calorimeter. Total body lipid content was measured by the Folch extraction method [12]. Total body protein content was determined using a formula relating total energy value of the carcass, energy derived from fat, and energy derived from protein [13]; the energy values for body fat and protein were taken as 39.2 and 23.5 kJ/g, respectively [14].

### Plasma concentrations of NEFA

The blood samples were centrifuged at 1400 g for 8 min at 4°C. Plasma was removed and NEFA were measured by colorimetric enzymatic method using commercial kits (Randox Laboratories ltd., United Kingdom).

### Preparation of isolated subsarcolemmal and intermyofibrillar skeletal muscle mitochondria

Hind leg skeletal muscles (gastrocnemius, soleus, tibialis anterior and quadriceps) were rapidly removed and used for preparation of isolated subsarcolemmal and intermyofibrillar mitochondria, as previously reported [15]. Briefly, skeletal muscles were homogenised in an isolation medium containing 100 mM KCl, 50 mM Tris, pH 7.5, 5 mM MgCl2, 1 mM EDTA, 5 mM EGTA and 0.1% (w/v) fatty acid-free bovine serum albumin (BSA). Aliquots of homogenates were withdrawn for measurement of oxygen consumption, and then the homogenate was centrifuged at 500 g for 10 min. The homogenate supernatant was centrifuged at 3000 g for 10 min and the resulting pellet, containing subsarcolemmal mitochondria, was washed twice, and resuspended in a suspension medium containing 250 mM sucrose, 50 mM Tris, pH 7.5, 0.1% (w/v) fatty acid free BSA. The homogenate precipitate was resuspended in a small amount of isolation medium and treated with protease (9 U/g tissue) for 5 min at a temperature of 4°C. The suspension was then homogenised, filtered through sterile gauze, and centrifuged at 3000 g for 10 min. The resulting supernatant was rapidly discarded and the pellet was resuspended and centrifuged at 500 g for 10 min. The supernatant containing the intermyofibrillar mitochondria was centrifuged at 3000 g for 10 min, the pellet was washed once and resuspended in the suspension medium. In control experiments, we assured that the differences in functionality of intermyofibrillar and subsarcolemmal mitochondria were not due to differences in isolation procedures. In fact, contamination of subsarcolemmal and intermyofibrillar mitochondria by other ATPase-containing membranes was lower than 10%, protease treatment of subsarcolemmal mitochondria had no effect on state 3 and 4 respiratory activities and addition of cytochrome c (3 nmol/mg protein) only enhanced state 3 respiration by approximately 20% and 10% in subsarcolemmal and intermyofibrillar mitochondria, respectively.

### Measurement of mitochondrial oxidative capacities, proton leak, lipid peroxidation and SOD specific activity

Oxygen consumption rate was measured polarographically with a Clark-type electrode (Yellow Springs Instruments, OH, USA) in a 3 ml-glass cell, at a temperature of 30°C. Skeletal muscle homogenates were incubated in a medium containing 30 mM KCl, 6 mM MgCl_2_, 75 mM sucrose, 1 mM EDTA, 20 mM KH_2_PO_4_ and 0.1% (w/v) fatty acid free BSA, pH 7.0 using 10 mM succinate + 3.8 μM rotenone as substrate and oxygen consumption was measured in the presence of 0.3 mM ADP (state 3) or 4 μg/ml oligomycin (state 4). Skeletal muscle isolated mitochondria were incubated in the same above medium using 10 mM succinate + 3.8 μM rotenone as substrate. After the addition of 0.3 mM ADP, state 3 oxygen consumption was measured, while state 4 was obtained from oxygen consumption measurements at the end of state 3, when ADP becomes limiting. Respiratory control ratio (RCR) was calculated as state 3/state 4 ratio.

If the activity of the respiratory chain is titrated with inhibitors in the presence of oligomycin to prevent ATP synthesis, the resulting titration curve of membrane potential against respiration rate represents the kinetic response of the proton leak to changes in membrane potential. Mitochondrial oxygen consumption was measured polarographically with a Clark-type electrode, whereas mitochondrial membrane potential recordings were performed in parallel with safranin O using a dual-wavelength spectrophotometer (511–533 nm) [16]. The absorbance readings were transformed into mV membrane potential using the Nernst equation: Δψ = 61 mV log ([K+]in/[K+]out). Calibration curves made for each preparation were obtained from traces in which the extramitochondrial K + level ([K+]out) was altered in the 0.1–20 mM range. The change in absorbance caused by the addition of 3 μM valinomycin was plotted against [K+]out. Then, [K+]in was estimated by extrapolation of the line to the zero uptake point. Titration of mitochondrial oxygen consumption and membrane potential were carried out at 30°C by sequential additions of increasing malonate concentrations, in a medium containing 30 mM LiCl, 6 mM MgCl2, 75 mM sucrose, 1 mM EDTA, 20 mM Tris-PO4 pH 7.0, succinate (10 mM), rotenone (3.75 μM), oligomycin (2 μg/ml), safranin O (83.3 nmol/mg), nigericin (80 ng/ml) and 0.1% (w/v) fatty acid-free BSA.

Lipid peroxidation in isolated subsarcolemmal and intermyofibrillar mitochondria was determined according to Fernandes *et al*. [17], by measuring thiobarbituric acid reactive substances (TBARS), using the thiobarbituric acid assay. Aliquots of mitochondrial suspensions were added to 0.5 ml of ice-cold 40% trichloroacetic acid. Then, 2 ml of 0.67% of aqueous thiobarbituric acid containing 0.01% of 2,6-di-tert-butyl-p-cresol was added. The mixtures were heated at 90°C for 15 min, then cooled in ice for 10 min, and centrifuged at 850 g for 10 min. The supernatant fractions were collected and lipid peroxidation was estimated spectrophotometrically at 530 nm. The amount of TBARS formed was calculated using a molar extinction coefficient of 1.56 × 10^5^/M/cm and expressed as nmol TBARS/mg protein.

SOD specific activity was measured in a medium containing 0.1 mM EDTA, 2 mM KCN, 50 mM KH2PO4 pH 7.8, 20 mM cythocrome c, 0.1 mM xanthine, and 0.01 units of xanthine oxidase. Determinations were carried out spectrophotometrically (550 nm) at 25°C, by monitoring the decrease in the reduction rate of cythocrome c by superoxide radicals, generated by the xanthine-xanthine oxidase system. One unit of SOD activity is defined as the concentration of enzyme that inhibits cythocrome c reduction by 50% in the presence of xanthine + xanthine oxidase, and the obtained values were normalized for mg of proteins [18].

### Western blot quantification of uncoupling protein 3 in isolated subsarcolemmal and intermyofibrillar skeletal muscle mitochondria

Samples were denatured in a buffer (60.0 mM Tris pH 6.8, 10% sucrose, 2% SDS, 4% β-mercaptoethanol) and loaded onto a 12% SDS-Polyacrylamide gel. After the run in electrode buffer (50 mM Tris, pH 8.3, 384 mM glycine, 0.1% SDS), the gels were transferred onto PVDF membranes (Millipore, MA, USA) at 0.8 mA/cm2 for 90 minutes. The membranes were preblocked in blocking buffer (PBS, 5% milk powder, 0.5% Tween 20) for 1 hour and then incubated overnight at 4°C with rabbit polyclonal antibody for UCP3 (Millipore, MA, USA, diluted 1:3000 in blocking buffer). Membranes were washed 3 times 12 minutes in PBS/0.5% Tween 20 and 3 times 12 minutes in PBS; and then incubated 1 hour at room temperature with alkaline phosphatase-conjugated secondary antibody (Promega, WI, USA). The membranes were washed as above described, rinsed in distilled water and incubated at room temperature with a chemiluminescent substrate, CDP-Star (Sigma-Aldrich, MO, USA). Data detection was carried out by exposing autoradiography films (Eastman Kodak Company, NY, USA) to the membranes. Quantification of signals was carried out by Un-Scan-It gel software (Silk Scientific, UT, USA). Values were expressed as optical density arbitrary units and were normalized for mg of proteins. Equal loading was verified by Ponceau S staining.

### Statistical analysis

Data are given as means ± SEM. Statistical analyses were performed by non parametric Mann Whitney test, non parametric two-way ANOVA [19] or non linear regression curve fit. Probability values less than 0.05 were considered to indicate a significant difference. All analyses were performed using GraphPad Prism 6 (GraphPad Software, CA, USA).

### Materials

All chemicals used were of analytical grade and were purchased from Sigma (St. Louis, MO, USA).

## Results

### Animal metabolic characterization

Body composition analysis reveals that old rats displayed not only higher body weight, but also significantly higher body energy and lipids, while body proteins were significantly lower, compared to adult rats (Table 1). In addition, plasma NEFA were significantly higher in old than in adult rats, while RMR were found to be significantly lower in old rats compared to adult ones, both in fed and fasting conditions (Table 1).

**Table 1:** Body composition, plasma non esterified fatty acids and resting metabolic rate in adult and old rats

	**Adult**	**Old**
Body weight, g	492 ± 10	598 ± 26*
Body energy, kJ/100 g	1010 ± 24	1098 ± 21*
Body lipids, kJ/100 g	650 ± 20	862 ± 23*
Body proteins, kJ/100 g	374 ± 22	240 ± 10*
Plasma non esterified fatty acids, mM	0.49 ± 0.05	0.96 ± 0.04*
Fed resting metabolic rate, μmol oxygen/(min x g protein)	9.3 ± 0.2	7.4 ± 0.2*
Fasting resting metabolic rate, μmol oxygen/(min x g protein)	8.0 ± 0.2	6.0 ± 0.2*

Values are the means ± SEM of eight different rats. *P < 0.05 compared to adult rats.

### Aging induces a decrease in skeletal muscle mitochondrial oxidative capacity

Oxidative capacities were assessed in whole tissue homogenates and in intermyofibrillar and subsarcolemmal skeletal muscle isolated mitochondria and significantly lower values were found in old rats compared to adult ones (Table 2).

**Table 2:** Oxidative capacities in homogenates, intermyofibrillar and subsarcolemmal skeletal muscle mitochondria from adult and old rats

	**Adult**	**Old**
Whole Tissue Homogenate		
ngatoms oxygen/(min x g wet tissue)		
state 3	4520 ± 220	3590 ± 185*
state 4	779 ± 48	630 ± 50*
RCR	5.8 ± 0.2	5.7 ± 0.2
Intermyofibrillar Mitochondria		
ngatoms oxygen/(min x mg protein)		
state 3	756 ± 48	577 ± 48*
state 4	131 ± 8	101 ± 10*
RCR	5.8 ± 0.1	5.7 ± 0.1
Subsarcolemmal Mitochondria		
ngatoms oxygen/(min x mg protein)		
state 3	434 ± 20#	370 ± 20#*
state 4	83 ± 4#	73 ± 6#*
RCR	5.2 ± 0.1	5.1 ± 0.1

Values are the means ± SEM of eight different rats. *P < 0.05 compared to adult rats; #P < 0.05 compared to intermyofibrillar mitochondria (non parametric two-way ANOVA). RCR = respiratory control ratio.

### Aging is characterized by decreased proton leak in skeletal muscle mitochondria

**Figure 1:**
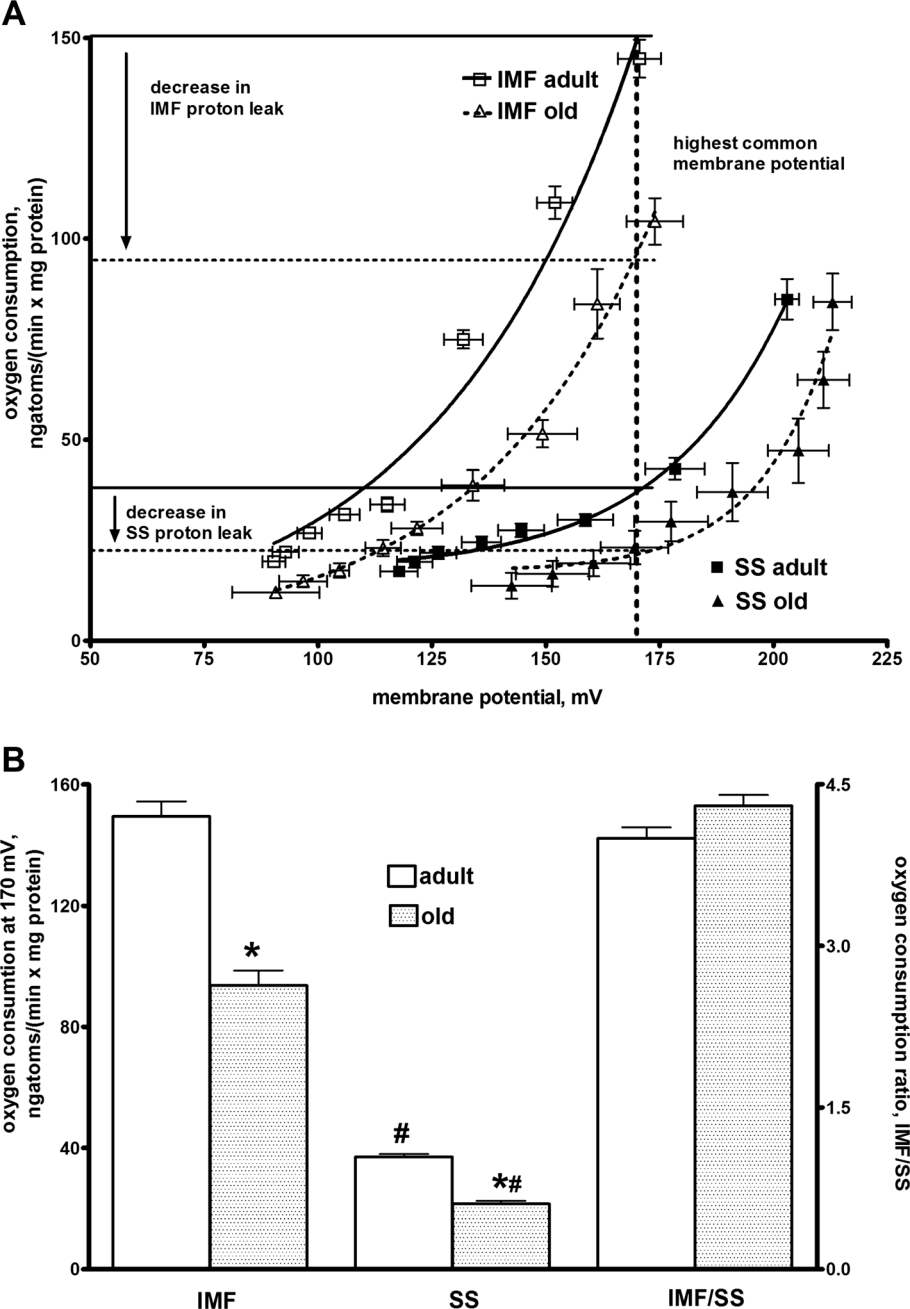
**Proton leak in muscle mitochondria from adult and old rats. A**: Titration curve of membrane potential against respiration rate during state 4 respiration represents the kinetic response of the proton leak to changes in membrane potential in intermyofibrillar and subsarcolemmal skeletal muscle mitochondria. Non linear regression curve fits show that proton leak was significantly (P < 0.05) lower in subsarcolemmal than in intermyofibrillar mitochondria, and in intermyofibrillar and subsarcolemmal mitochondria from old rats compared to adult rats. **B**: Oxygen consumption values at the highest common membrane potential (170 mV) and ratio between intermyofibrillar and subsarcolemmal oxygen consumption values at 170 mV in skeletal muscle from adult and old rats. Values are the means ± SEM of eight different rats. IMF = intermyofibrillar; SS = subsarcolemmal. *P < 0.05 compared to adult rats; ^#^P < 0.05 compared to intermyofibrillar mitochondria (non parametric two-way ANOVA).

Indirect estimate of proton leak of mitochondrial inner membrane was obtained by simultaneous measurement of mitochondrial membrane potential and oxygen consumption during malonate titrations of electron transport chain, since steady state oxygen consumption rate (i.e. proton efflux rate) in non-phosphorylating mitochondria is equivalent to proton influx rate due to proton leak. The resulting curves for intermyofibrillar and subsarcolemmal mitochondria from adult and old rats are presented in Figure 1A and show that the oxygen used to balance the leak over a range of membrane potential is lower (i.e., the leak is lower) in subsarcolemmal than in intermyofibrillar mitochondria both in adult and old rats, and the oxygen used to balance the leak over a range of membrane potential is lower (i.e., the leak is lower) in both mitochondrial populations in old rats. Statistical comparison of oxygen consumption at the highest common membrane potential shows that values obtained for old rats were significantly lower than those obtained for adult rats, both in subsarcolemmal and intermyofibrillar mitochondria (Figure 1B). In addition, values of oxygen consumption at the highest common membrane potential obtained for subsarcolemmal mitochondria were significantly lower than those found in intermyofibrillar mitochondria both in adult and old rats (Figure 1B), so that ratio between intermyofibrillar and subsarcolemmal values was about 4 both in adult and old rats (Figure 1B).

### Aging selectively causes oxidative damage in subsarcolemmal mitochondria

**Figure 2:**
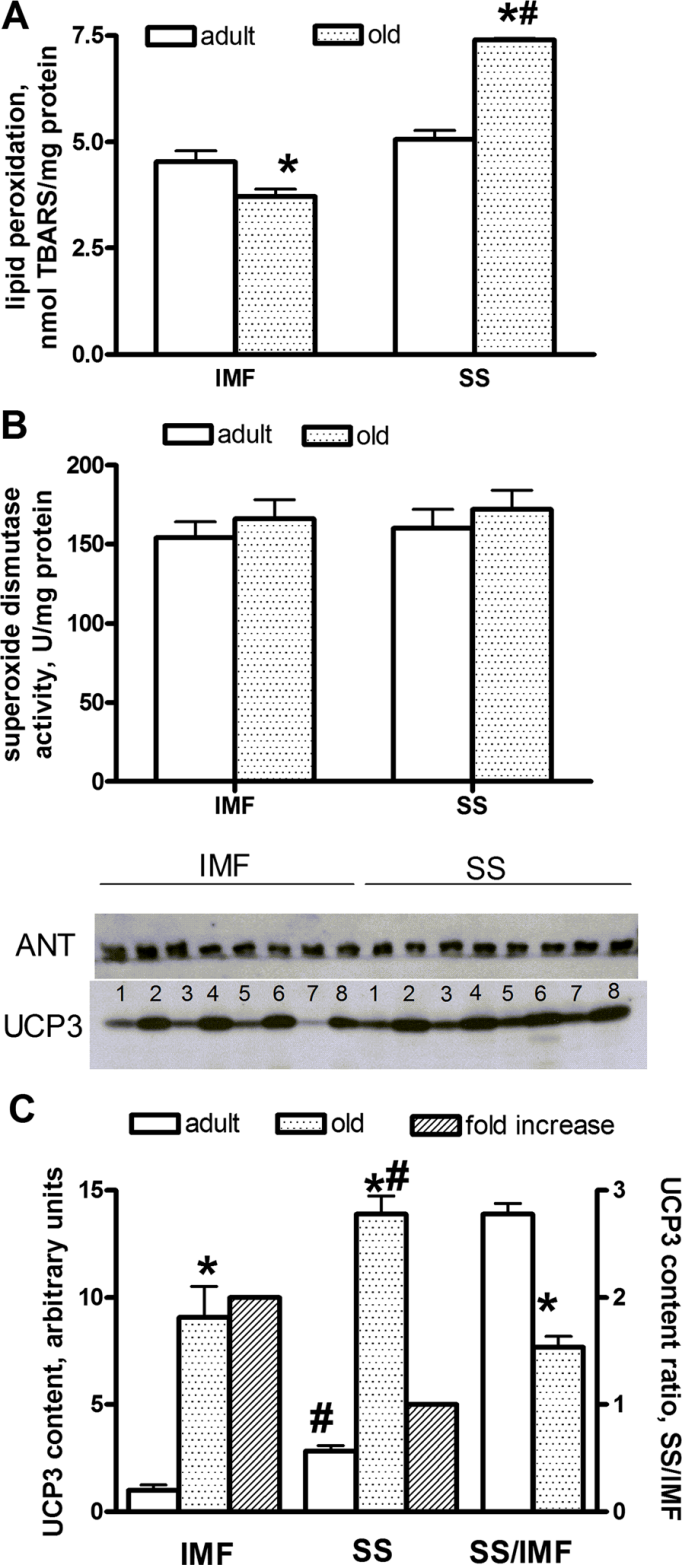
**Lipid peroxidation, superoxide dismutase and UCP3 content in muscle mitochondria from adult and old rats. A**: Lipid peroxidation in intermyofibrillar and subsarcolemmal skeletal muscle mitochondria from adult and old rats. **B**: Superoxide dismutase specific activity in intermyofibrillar and subsarcolemmal skeletal muscle mitochondria from adult and old rats. **C**: Quantification of UCP3 (with representative western blots; odd numbers = adult rats, even numbers = old rats) and UCP3 content ratio in intermyofibrillar and subsarcolemmal skeletal muscle mitochondria from adult and old rats. Values are the means ± SEM of eight different rats. IMF = intermyofibrillar; SS = subsarcolemmal; TBARS = thiobarbituric acid reactive substances. *P < 0.05 compared to adult rats; ^#^P < 0.05 compared to intermyofibrillar mitochondria (non parametric two-way ANOVA).

Lipid peroxidation was found to be similar in intermyofibrillar and subsarcolemmal mitochondria from adult rats (Figure 2A). Significantly lower values were found in intermyofibrillar skeletal muscle mitochondria from old rats, compared to adult ones, while values observed in subsarcolemmal mitochondria from old rats were significantly higher compared to those from adult rats, so that in old rats oxidative damage in subsarcolemmal mitochondria was higher than that found in intermyofibrillar ones (Figure 2A; IMF adult = 4.53 ± 0.20; IMF old = 3.70 ± 0.10; SS adult = 5.00 ± 0.20; SS old = 7.41 ± 0.04 nmol/mg protein). SOD specific activity, taken as an index of antioxidant defense, was not significantly affected in old rats (Figure 2B; IMF adult = 154 ± 10; IMF old = 166 ± 12; SS adult = 160 ± 12; SS old = 172 ± 12 U/mg protein). Western blot quantification of UCP3 in intermyofibrillar and subsarcolemmal skeletal muscle mitochondria revealed that subsarcolemmal mitochondria contained more UCP3 than intermyofibrillar ones, both in adult and old rats (Figure 2C). In addition, significantly higher content of this protein was found in both mitochondrial populations from old rats compared to adult ones (Figure 2C), even though the fold increase in UCP3 content in intermyofibrillar mitochondria was higher than that in subsarcolemmal mitochondria, so that ratio between subsarcolemmal and intermyofibrillar values significantly decreased in old rats (Figure 2C).

## Discussion

Our present results indicate that in old rats a lowered RMR is associated with increased energetic efficiency in intermyofibrillar and subsarcolemmal mitochondria from skeletal muscle, while oxidative damage is present only in subsarcolemmal mitochondria.

Whole body metabolic characterization shows that old rats display increased body weight and lipids, while protein mass is significantly lower. The reduced body proteins are indicative of a loss of metabolically active body mass, in agreement with the progressive loss of muscle mass and strength that characterizes aging [20]. In addition, the significant decrease in RMR normalized to protein mass found in old rats, both in fed and fasting conditions, indicates that additional mechanisms of energy saving are induced with advancing age. In laboratory rats, that display a sedentary behavior due to standard stabulation conditions, energy saving must occur in organs and tissues with high metabolic activity, such as skeletal muscle, that accounts for about 30% of whole body energy expenditure [21].

At the cellular level, mitochondria are the main site involved in the regulation of energy utilization. Therefore, it is very likely that the decreased RMR, found in old rats, could be linked to changes in the functionality of the two mitochondrial populations that are present in skeletal muscle and that display different activity and differential regulation in response to physiological stimuli [6,7]. Mitochondrial function depends on the activity of the respiratory chain complexes, as well as on the degree of coupling of oxidative phosphorylation. In fact, oxygen consumption and ATP synthesis are not completely coupled, due to the presence of a proton leak pathway that dissipates part of the energy coming from the oxidation of energy substrates [22]. Our present results show that, similarly to what previously found in younger rats [10], intermyofibrillar mitochondria display higher oxidative capacities than subsarcolemmal ones, both in adult and old rats. In addition, the significant decrease in oxidative capacity, found in subsarcolemmal and intermyofibrillar mitochondria from old rats, suggests an impairment in respiratory chain activity. This impairment is also evident when measurements are carried out in skeletal muscle homogenates, so that whole tissue oxidative capacity is blunted. The degree of reduction of oxidative capacity is similar in homogenates and isolated mitochondria (about -20%), and the RCR values of isolated subsarcolemmal and intermyofibrillar mitochondria are similar to those obtained in whole tissue homogenates, so we can conclude that the isolation procedure does not affect our present findings. Since oxidative capacity measured in the homogenate reflects the product of mitochondrial mass and activity, the similar age-induced decrease in oxidative capacity in homogenates and isolated mitochondria found in old rats suggests no change in mitochondrial mass. A decrease in mitochondrial biogenesis probably occurs with advanced aging, since a decrease in mitochondrial mass has been found in senescent rats (36 months old) [9]. Alternatively, reduced mitochondrial biogenesis could take place selectively in specific muscles, such as gastrocnemius, whose mitochondrial mass has been found decreased in old rats [23,24].

The significant decrease in state 4 respiration, here found, could indicate a decrease in the proton leak pathway. So, to assess whether aging is associated with changes in mitochondrial inner membrane permeability, we evaluated the relationship between membrane potential and oxygen consumption under state 4 condition. We found that, at the highest common membrane potential, the oxygen consumption is lower in subsarcolemmal than in intermyofibrillar mitochondria both in adult and old rats, and it is lower in subsarcolemmal and intermyofibrillar mitochondria from old rats, compared to adult ones. Therefore, mitochondrial proton leak is lower in subsarcolemmal mitochondria, compared to intermyofibrillar ones, and significantly decreases with aging in both mitochondrial populations. This decrease in proton leak implies an increase in mitochondrial degree of coupling, in agreement with results obtained in vivo in aged rat skeletal muscle and showing a trend for a higher coupling efficiency [24]. The lower proton leak is of relevance for skeletal muscle energy metabolism, since skeletal muscle cells in sedentary laboratory rats operate at most in conditions near to state 4, when the contribution of proton leak to total oxygen consumed by mitochondria is high [25]. In addition, in resting conditions, about 30% of whole body energy expenditure is due to skeletal muscle metabolism. Therefore, our present results strongly suggest that the increased mitochondrial coupling in skeletal muscle contributes to the decreased RMR here found and could theoretically explain the progression of age-induced obesity.

We also assessed oxidative damage, as well as SOD activity and UCP3 content, of the two skeletal muscle mitochondrial populations in adult and old rats. In fact, mitochondrial energetic efficiency influences oxidative stress, since it is well known that reactive oxygen species (ROS) production increases when mitochondrial potential is higher [26,27]. In addition, the degree of oxidative damage also depends on the ability to counteract ROS damage by antioxidant systems, such as SOD activity. Finally, UCP3 plays a role in the defence of mitochondria from oxidative damage, by translocating fatty acid peroxides from the inner to the outer membrane leaflet and preserving macromolecules from being oxidized by very aggressive fatty acid peroxides [11,28]. This mechanism could be particularly relevant when cellular fatty acid availability increases, such as in conditions of increased plasma NEFA levels. Firstly, in adult rats we found that UCP3 content is higher in subsarcolemmal mitochondria, that are more prone to ROS damage, due to their lower proton leak. As a result, when comparing the degree of lipid peroxidation in the two mitochondrial populations, we found no difference in adult rats. In addition we found a significant increase in UCP3 content in subsarcolemmal and intermyofibrillar mitochondria from old rats, but the UCP3 increase is 10 fold in intermyofibrillar mitochondria and only 5 fold in subsarcolemmal mitochondria, notwithstanding the fact that the decrease in proton leak is the same in both mitochondrial populations. The lower increase in UCP3 content in subsarcolemmal mitochondria is probably the cause of the higher oxidative damage found in this mitochondrial population. On the other hand, intermyofibrillar mitochondria are more protected by oxidative damage by the marked upregulation of UCP3, so preserving the capacity to produce ATP for muscle contraction. Further assessment of the degree of oxidative damage in the two skeletal muscle mitochondrial populations is required to support the above hypothesis. It is also possible that subsarcolemmal mitochondria are more prone to oxidative stress due to their localisation beneath the plasma membrane, where they probably experience higher NEFA concentration than intermyofibrillar ones, since NEFA dilute in the cytoplasm. It can be hypothesized that, with the progression of aging, the oxidative damage accumulates in subsarcolemmal mitochondria, causing loss of functional activity. This suggestion could explain the findings previously obtained in senescent (33–36 months old) rats, showing an increase in mitochondrial proton leak measured in the whole skeletal muscle mitochondrial population [29], or a decrease in mitochondrial membrane potential in subsarcolemmal but not in intermyofibrillar mitochondria [9].

The upregulation of UCP3 is also in line with the increased plasma NEFA, since it is well known that UCP3 synthesis is stimulated in all the conditions characterised by increased plasma NEFA, such as starvation, exercise, high fat diet [30]. It has been previously found that UCP3 was lower in white and unchanged in red gastrocnemius muscle fibres in old animals [31], but these results have been obtained on gastrocnemius muscle and could therefore not completely reflect the regulation of this protein with aging in the whole skeletal muscle tissue. In addition, these results, as well as those showing a decreased UCP3 content in skeletal muscle mitochondria from old rats [32,33], have been obtained using Fischer 344 rats, a rat strain that gain weight only moderately with age compared with other strains (i.e. Sprague–Dawley, Wistar, Long Evans). Thus, the regulation of UCP3 with aging in skeletal muscle mitochondria could be strain-dependent.

Taking all the above results as a whole, the modifications of mitochondrial function by us found in aging skeletal muscle could have deleterious consequences for whole body metabolic homeostasis. In fact, the increased mitochondrial coupling implies that less substrates need to be burned to obtain the same amount of ATP, so that more energy coming from the diet is stored in adipose tissue and age-induced obesity develops. The increased oxidative damage in subsarcolemmal mitochondria indicates that aging-induced impairment occurs mainly in this mitochondrial population, that is located beneath the plasma membrane and provides ATP for membrane transports and signal transduction pathways [34]. On the other hand, intermyofibrillar mitochondria, that provide ATP for muscle contraction, are less prone to oxidative damage. This could explain why it has been found that 2-year-old rats retain the capacity to increase skeletal muscle oxidative capacity and mitochondrial population density in response to endurance training [35].

## Conclusions

The increased energetic efficiency in intermyofibrillar and subsarcolemmal mitochondria from skeletal muscle found in old rats, which implies decreased energy expenditure, together with increased oxidative damage in subsarcolemmal mitochondria, if extrapolated to humans suggest dangerous metabolic consequences in sedentary old subjects.

## Abbreviations

NEFA: Non esterified fatty acids; RMR: Resting metabolic rate; SOD: Superoxide dismutase; UCP3: Uncoupling protein 3; ROS: Reactive oxygen species; RCR: Respiratory control ratio; BSA: Bovine serum albumin; TBARS: Thiobarbituric acid reactive substances.

## Competing interest

The authors declare that they have no competing interests.

## Authors’ contributions

SI designed and supervised the study. SI and GL obtained funding and provided administrative, technical and material support. RC, FB, AM and AG performed the animal experiments. RC and SI contributed to the analysis of data and interpretation of the results, RC, SI and GL wrote the draft of the manuscript, and all the authors critically reviewed the manuscript. All authors read and approved the final manuscript.

## Pre-publication history

The pre-publication history for this paper can be accessed here:

http://www.biomedcentral.com/1471-2318/14/79/prepub
